# Computed Protein–Protein Enthalpy Signatures as a Tool for Identifying Conformation Sampling Problems

**DOI:** 10.1021/acs.jcim.3c01041

**Published:** 2023-09-28

**Authors:** Süleyman Selim Çınaroğlu, Philip C. Biggin

**Affiliations:** Department of Biochemistry, University of Oxford, South Parks Road, Oxford OX1 3QU, U.K.

## Abstract

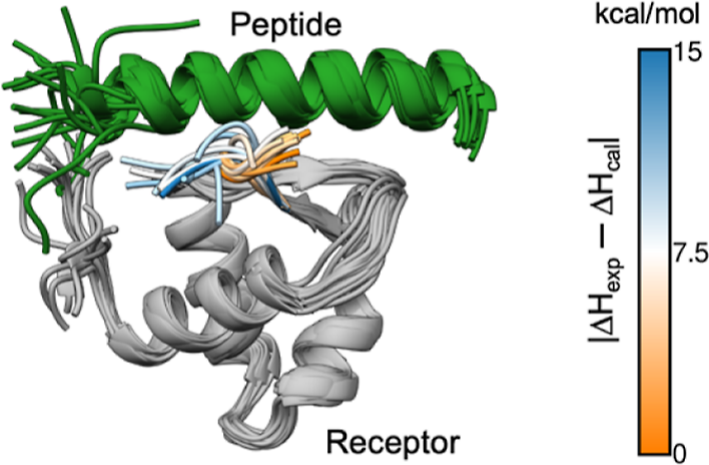

Understanding the thermodynamic signature of protein–peptide binding events is a major challenge in computational chemistry. The complexity generated by both components possessing many degrees of freedom poses a significant issue for methods that attempt to directly compute the enthalpic contribution to binding. Indeed, the prevailing assumption has been that the errors associated with such approaches would be too large for them to be meaningful. Nevertheless, we currently have no indication of how well the present methods would perform in terms of predicting the enthalpy of binding for protein–peptide complexes. To that end, we carefully assembled and curated a set of 11 protein–peptide complexes where there is structural and isothermal titration calorimetry data available and then computed the absolute enthalpy of binding. The initial “out of the box” calculations were, as expected, very modest in terms of agreement with the experiment. However, careful inspection of the outliers allows for the identification of key sampling problems such as distinct conformations of peptide termini not being sampled or suboptimal cofactor parameters. Additional simulations guided by these aspects can lead to a respectable correlation with isothermal titration calorimetry (ITC) experiments (*R*^2^ of 0.88 and an RMSE of 1.48 kcal/mol overall). Although one cannot know prospectively whether computed ITC values will be correct or not, this work shows that if experimental ITC data are available, then this in conjunction with computed ITC, can be used as a tool to know if the ensemble being simulated is representative of the true ensemble or not. That is important for allowing the correct interpretation of the detailed dynamics of the system with respect to the measured enthalpy. The results also suggest that computational calorimetry is becoming increasingly feasible. We provide the data set as a resource for the community, which could be used as a benchmark to help further progress in this area.

## Introduction

Protein–protein interactions play a vital role in many biological processes and underpin cellular signal transduction events within (and beyond) the cell.^1^ A deeper understanding of the interactome will be necessary to fully appreciate the role of protein–protein interactions in pathology^2^ and will aid in the identification of novel targets for therapeutic intervention. The need for safer, more effective medicines is more urgent than ever.^3^ Protein–protein interactions are a promising and rapidly growing area of drug discovery, with important implications for treating many diseases.^4^

Understanding the principles of protein–protein association requires a comprehensive description of the thermodynamic and kinetic characteristics of binding.^5^ In this work, we focus solely on the thermodynamic aspects. Identifying the driving forces that stabilize the interaction has been actively pursued for decades.^6,7^ Protein–protein binding is a complicated process, including hydrophobic, van der Waals, and electrostatic interactions. Three-dimensional structures of protein–protein complexes can be obtained using X-ray crystallography, NMR spectroscopy, and cryo-electron microscopy, which provide valuable information on critical interactions between binding site residues for an atomic-level understanding of binding mechanisms.^8^ Alongside the structural efforts, calorimetric approaches that provide thermodynamic signatures of binding give complementary insight into the forces that stabilize protein–protein complexes.^9^

While there are many techniques for obtaining binding affinity, isothermal titration calorimetry (ITC) can sensitively measure the enthalpic (Δ*H*) component, and thus, if Δ*G* is known, then the entropy (Δ*S*) can be also derived to provide a full thermodynamic “signature” for a binding event. However, the quantitative prediction of binding affinities (and indeed the underlying ensembles of structures that give rise to it) remains a significant challenge for computational chemistry.^10,11^ Surmounting this challenge would aid our understanding of regulation in biological systems and also improve our prospects for drug design and development.

The development of computing architecture and new algorithms has led to a surge in the use of molecular dynamics (MD) simulation tools over recent decades.^12^ MD simulations can provide unique insight into the behavior of molecules and their interactions at atomic resolution. Using these simulations, researchers can also estimate thermodynamic quantities to gain knowledge about microscopic details and relate thermodynamic measurements to physical interactions. The advancement of computational methods for estimating binding free energy is well documented.^13^ However, the prediction of the Δ*S* and Δ*H* components of binding remains more complicated. Estimating the Δ*S* and Δ*H* components is generally less accurate than estimating the binding free energy itself. The direct method of estimating enthalpy is the most straightforward approach and allows a direct explanation of the physical forces involved.^14^ However, the direct method relies on the difference between the energies of the bound and unbound states calculated using four separate simulations.^15^ Furthermore, the reliability of the direct method depends on sufficient sampling that can realistically only be obtained with a huge amount of simulation data.^11^

In this work, we set out to explore two aspects of protein–protein enthalpy. First, we wanted to know what kind of performance one could expect with a typical force field. Second, and in anticipation of outliers, what conclusions could be drawn from them about the sampling of the ensemble? To do this, we compute the absolute binding enthalpy from MD simulations of a diverse set of protein–protein complexes using the direct method with the multibox approach. Although the theory has been around for some time, there are very few reports of its application to anything more than host–guest systems, presumably because researchers have assumed that the sampling and consequent error would make the results too difficult to interpret. We show here that although it is still extremely challenging to prospectively predict enthalpy, the (lack of) agreement between experiment and prediction can be used as an indicator of when conformational sampling may not be reflective of the correct ensemble–perhaps as a result of being stuck in a metastable state, which by many metrics could give the illusion of complete sampling. Finally, the data presented here could serve as a benchmark for future studies.

## Methods

### Building a Nonredundant Data Set

To investigate how well protein–protein enthalpy can be predicted, we first obtained all protein–protein complexes from the PDBbind v2020.^16^ In all cases, the chain with the longest sequence length was defined as the receptor, while the short chain was defined as the peptide for each PDB entry. PDB entries having receptors with sequences longer than 150 residues were eliminated to reduce the computational cost in MD simulations. The remaining PDB entries (736) were then investigated as to whether they had corresponding binding enthalpy data obtained by ITC associated with them in the literature. This resulted in 76 PDBs.

We then employed two different clustering approaches to reduce the redundancy in the data. First, we extracted receptor amino acid sequences from RCSB PDB,^17^ then the amino acid sequences were aligned using Clustal omega^18^ and clusters were defined by the result of their phylogenetic relationships of the sequences. Second, a sequence-independent clustering based on the local backbone similarity matching was performed using the MaxCluster tool for receptors.^19^ The final data set was selected by choosing the lowest number of total residues of the protein–peptide system from each cluster, with a view to reducing computational cost as far as possible. We also paid attention to the existence of complete ITC details and the same temperature (25 °C) when choosing the representatives. The final data set is composed of 11 complexes.

### MD Simulation Setup

The PDB structures were obtained from the RCSB PDB database for the data set (Table 1). Missing atoms and loops were modeled with the Modeler implemented in UCSF Chimera,^20^ and all heteroatoms were removed from the system except all crystallographic waters. We used the AMBER ff14SB force field for the protein and the TIP3P water model for water molecules.^21^ The zinc AMBER force field (ZAFF) was additionally used for Zn^2+^ atoms parameterization in the PHD zinc finger of BAZ2A (PDB: 4Q6F),^22,23^ while default parameters in ff14SB were used for Ca^2+^ atoms in calmodulin (PDB: 2LQC).

**Table 1:** Nonredundant Data Set for Testing Binding Enthalpies of Protein–Peptide Systems

PDB	cluster ID^a^	receptor^b^	peptide^b^	Δ*H* (kcal/mol)^c^	refs
1DPU	7(1)	P15927_(201–272)_	P13051_(66–91)_	–16.80 ± 0.28	Xie et al^32^
1RST	4(1)	P22629_(39–163)_	AWRHPQFGG	–12.56 ± 0.09	Schmidt et al^33^
2LQC	8(5)	P0DP23_(1–77)_	Q13936_(45–68)_	–6.91 ± 0.07	Liu and Vogel^34^
2MNU	8(1)	P02751_(5–83)_	APT_(201–226)_	–4.60 ± 0.10	Yu et al^35^
2MWY	5(6)	O15151_(23–111)_	P04637_(15–29)_	–17.50 ± 0.30	Grace et al^36^
4F14	9(3)	O76041_(955–1014)_	A4UGR9_(2245–2258)_	–8.46 ± 0.48	Eulitz et al^37^
4Q6F	11(4)	Q9UIF9_(1673–1728)_	P68431_(1–9)_	–9.81 ± 0.04	Tallant et al^38^
5E0M	10(8)	Q24117_(410–478)_	Q9H4H8_(434–446)_	–8.20 ± 0.10	Clark et al^39^
5OVC	2(2)	Q9JLU4_(570–664)_	P97836_(986–992)_	–6.80 ± 0.04	Ponna et al^40^
6EVO	3(9)	O15460_(142–236)_	PPGPRGPPG	–8.70 ± 0.80	Murthy et al^41^
6H8C	6(7)	P60520_(3–117)_	Q9GZZ9_(337–350)_	–5.91 ± 0.09	Huber et al^42^

a Cluster IDs from sequence-based clusters with structure-based cluster IDs in parentheses. As cluster 1 from sequence-based clustering was a single structure, this was omitted.

b UniProt IDs for receptors and peptides; some peptides are shown as their amino acid residues.

c Experimental binding enthalpy values.

All simulations were run using the Gromacs v2020 software package.^24^ A 3-step steepest descent energy minimization with a maximum force of 10 kJ/mol/nm^2^ was applied to all systems.^25^ In the first step, position restraints with a harmonic potential with a force constant of 1000 kJ/(mol/nm^2^) were applied to all heavy atoms, followed by a second step with position restraints only on the solute heavy atoms and then a final step with no restraints.

*NVT* and *NPT* ensemble simulations for 1 ns were performed to equilibrate all systems with positional restraints with the harmonic potential at a force constant of 1000 kJ/(mol/nm^2^) on heavy atoms of protein and ligand. Additionally, another *NPT* ensemble simulation for 1 ns was performed without restraints before the production run for data collection. The V-rescale^26^ and Parrinello and Rahman^27^ algorithms equilibrated the temperature at 300 K and the pressure at 1.0 bar, respectively. Unbonded interactions were calculated up to a cutoff of 1.0 nm with a potential shift. A dispersion correction was applied to the energy and pressure. All H-bond lengths were constrained with a LINear Constraint Solver (LINCS) algorithm.^28^ Coulomb interactions were evaluated with the fast smooth particle-mesh Ewald electrostatics method with an initial short-range cutoff of 1.0 nm.^29^ The leapfrog algorithm was used to run 20 independent 100 ns MD simulations with a 2 fs time step. Snapshots from the production runs were taken every 100 ps for 3D coordinates, while snapshots were taken every 100 fs for energy data.

### Absolute Binding Enthalpy Calculations

The binding enthalpy (Δ*H*) is calculated by eq 1, where ⟨*E*⟩_complex_, ⟨*E*⟩_solvent_, ⟨*E*⟩_receptor_, and ⟨*E*⟩_ligand_ are the averaged potential energies of the system from four separate simulations. In this method, the number of atoms between the bound and unbound states of the complex should exactly balance. Note that the pressure–volume contribution for the binding enthalpy is negligible.^15^
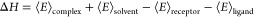
1where ⟨···⟩ is the time-averaged value obtained from multiple independent MD trajectories. The use of multiple simulations can provide a broader sampling of conformational space than a single long simulation. Thus, the trajectories allow the determination of mean values distributed across the potential energy surface.

The mean energy for each trajectory is estimated by averaging over the time period corresponding to the *N* number of snapshots, while the expected energy value for the set of trajectories, ⟨*E*⟩, is determined by calculating the means of the individual trajectories. For *K* number of trajectories, the ensemble mean is
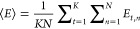
2where *E*_*t*,*n*_ is the energy value of the *n*’th snapshot of the *t*’th trajectory. *K* is the set of 20 trajectories for complex and apo-receptor simulations, while 10 trajectories for peptide in water and water-only simulations, and *N* (1,000,000) is defined as the number of snapshots taken from a 100 ns trajectory. Convergence of the potential energy was evaluated by plotting the cumulative average of the simulation data while the uncertainty [standard error of the mean (SEM)] in the energy values obtained from *K* independent trajectories was estimated using reblocking analysis,^30^ as implemented in the pyblock tool (https://pyblock.readthedocs.io). The SEM of Δ*H* is given by
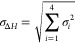
3where σ_*i*_ obtained from the reblocking analysis is the SEM of the overall potential energy of each system (complex, water, receptor, and peptide) because Δ*H* is an additive combination of mean energies. The main obstacle in interpreting the reblocking analysis is the choice of an optimal block size, giving the true SEM. A clear plateau in the reblock plot of the SEM versus block size ideally represents the SEM. However, it is neither objective nor efficient to individually analyze the reblock plot for each calculation to make decisions. It is possible to select an optimal block size via the use of eq 4
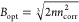
4where *n* is the number of data points and *n*_corr_ is the correlation length described elsewhere.^31^ However, to err on the side of caution, in this study, we took the maximum SEM value from each reblocking analysis.

## Results

### Obtaining a Nonredundant Data Set for Protein–Protein Enthalpy Calculations

**Figure 1:**
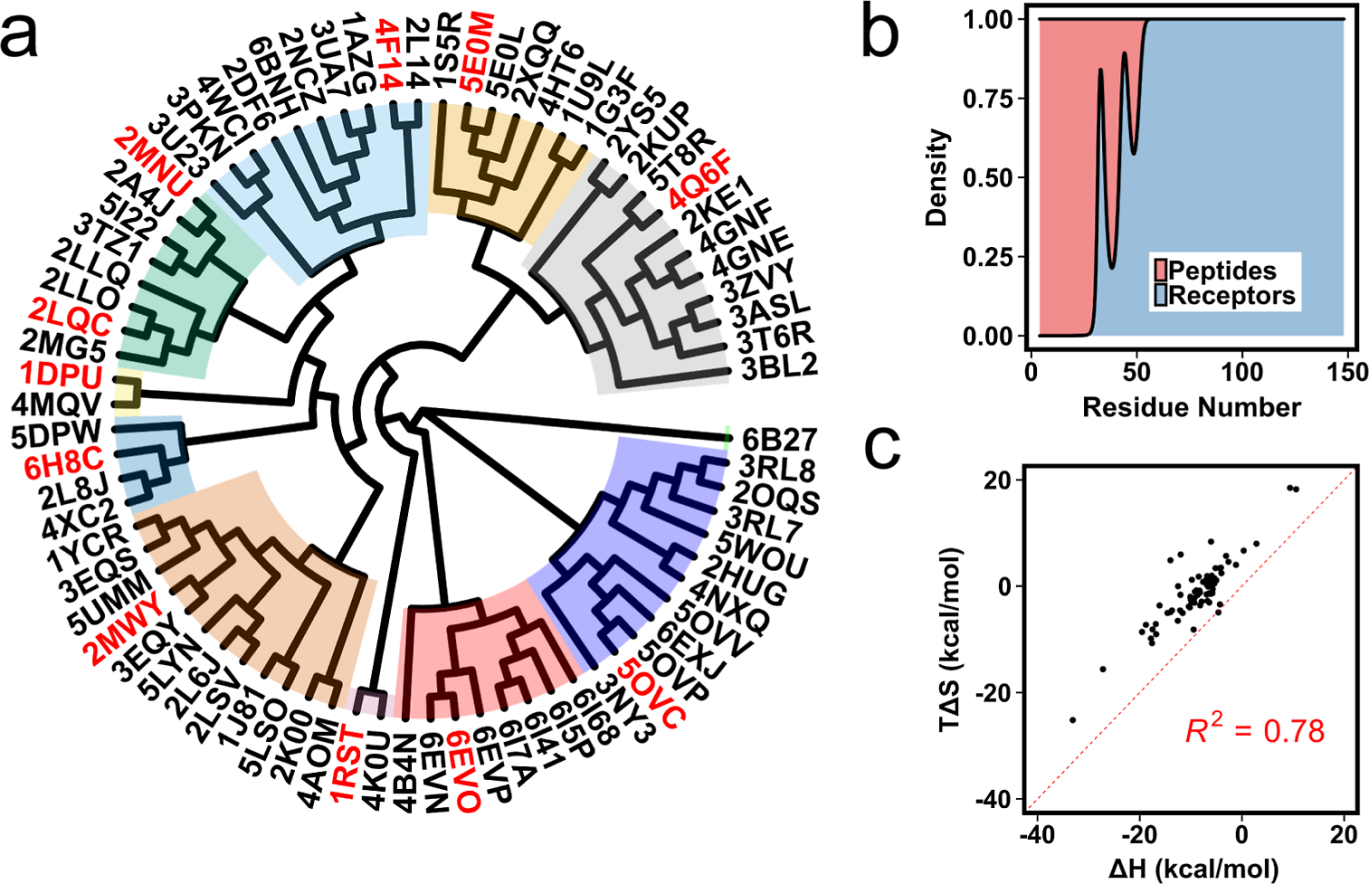
(a) Phylogenetic tree of receptor sequences from 76 PDBs. Red PDB IDs are the ones used for the final benchmark. (b) Density distribution for sequence lengths of peptides and receptors. (c) Entropy–enthalpy scatter plot for the 76 protein–protein complexes.

We extracted 76 PDB entries where the larger of the protein–peptides in the complex was smaller than 150 residues and where binding enthalpy data were available in the literature (Figure 1a and Supporting Information). The data set contains 25 NMR and 51 X-ray crystal structures with a resolution ranging from 1.11 to 2.6 Å. Receptor proteins come from 49 different genes of 12 organisms including two virus genomes. The receptor size ranges from 54 to 148 residues, while the peptides range from 4 to 49 residues (Figure 1b).

After obtaining PDB entries having binding enthalpies, we performed clustering based on the sequence and structure of the receptors. First, we ran Clustal Omega, a multiple-sequence alignment program, for receptor sequences and obtained the phylogenetic tree of the receptor sequences. The tree-based clustering gave us 11 different clusters with cluster sizes ranging from 1 to 11 (Figure 1a). 6B27 was the only member of cluster 1, however, it is a tandem repeat of SH3 domains, which mostly exist in cluster 9.

Indeed, closer inspection revealed that some domains (e.g., SH3 and EF-Hand) were found in different clusters derived from the sequence-based clustering process, and thus, we decided to use a structure-based clustering via the MaxCluster tool (http://www.sbg.bio.ic.ac.uk/maxcluster/) using the local backbone similarity matching method. Structure-based clustering gave us eight different clusters, with cluster sizes ranging from 4 to 12 members. We also had an additional cluster for all other PDBs having unrelated structures that are not similar (cluster ID 1—see Table 1).

Finally, we picked representative PDBs with the smallest number of total residues and complete ITC that were all performed at the same temperature (25 °C), giving us 11 different complexes in the data set (Table 1), which represent the clusters obtained from both methods. Cluster 1 obtained by structure-based clustering is equivalent to the three different clusters via the sequence-based method.

### Initial Results for Absolute Binding Enthalpy Calculations

**Figure 2:**
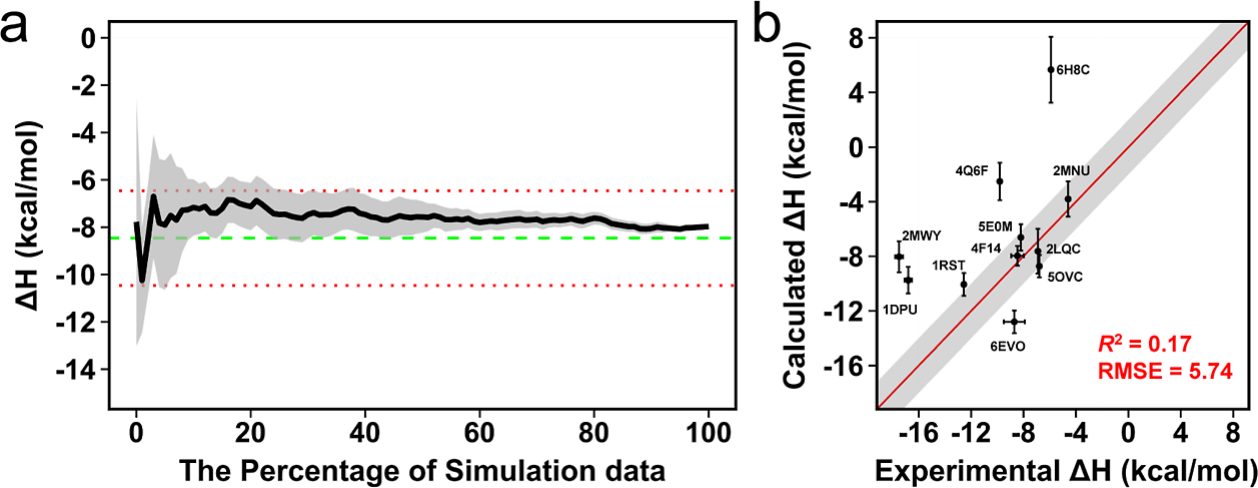
(a) Convergence pattern of the calculated Δ*H* by using 60 trajectories for the protein–peptide complex in PDB:4F14, giving good agreement with the experimental Δ*H* value. The green dashed line is the experimental Δ*H* and the red dotted lines indicate the 2 kcal/mol error limit. (b) Comparison of calculated binding enthalpies from experimental values. Error bars were drawn by using the highest SEM values from the reblocking analysis. The line of equivalence is shown in red, and the black shadow indicates the 2 kcal/mol error limit.

To the best of our knowledge, there has been no previous attempt to compute binding enthalpies for protein–protein systems using MD simulations. Thus, in the first instance, we wanted to evaluate just how well a standard approach would perform. Here, the absolute binding enthalpy for 11 protein–peptide complexes was estimated by the direct method (eq 1) using ensemble averaging (eq 2). In obtaining binding enthalpies, sufficient sampling to cover all conformational space is crucial to obtaining a converged pattern of potential energy. In previous studies, Roy et al. performed multiple independent simulations,^14^ while Li and Gilson ran long simulations to get sufficient sampling for the relative binding enthalpy calculation of a protein–ligand system.^11^ Here, we opted to perform multiple independent simulations to maximize sampling as we previously obtained good results for bromodomain–ligand complexes using this strategy.^43^ Thus, 60 trajectories, totaling 6 μs of simulation data, were generated from MD simulations of the bound and unbound states for each protein–peptide complex. We produced one million snapshots per simulation for the potential energy, which means 60 million data points were used for each Δ*H* calculation. Before performing enthalpy calculations, we first checked the overall convergence pattern for each complex. The cumulative convergence in increasing blocks of all simulation data shows a reasonably well-converged pattern of the calculated Δ*H*, as exemplified here for 4F14 (Figure 2a). This pattern was almost the same with most of the protein–peptide complexes (Figure S1). However, 2LQC and 6H8C exhibited a rather uneven profile, indicating that these complexes require more simulation data for convergence. Nevertheless, 2LQC gave a highly accurate result, but 6H8C gave the worst result (Figure 2b). Overall, the correlation with the experiment was poor, with an *R*^2^ = 0.17, and the accuracy of the calculations was weak with an average of RMSE = 5.74 kcal/mol. The results for only 5 PDB complexes are within the 2 kcal/mol error limit.

### Tail Conformations Affect the Accuracy of Predictions

**Figure 3:**
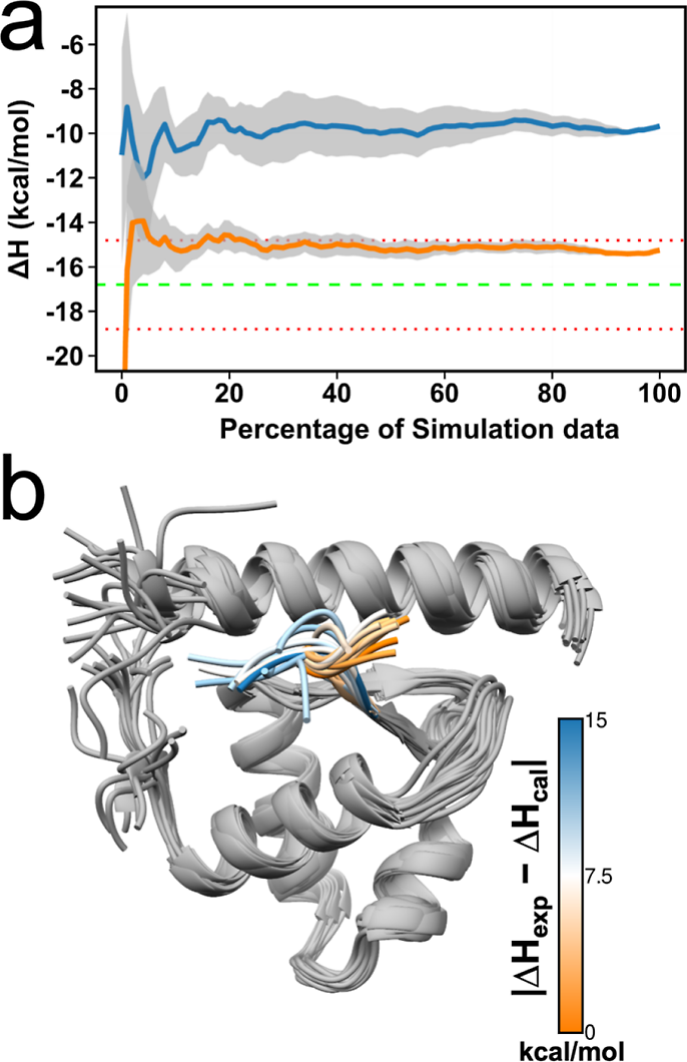
(a) Convergence pattern of the calculated Δ*H* by using 60 trajectories for two simulation setups for the RPA32–UNG2 complex. The blue line shows calculated Δ*H* for the first simulation set, while the orange line shows for the second simulation set. The green dashed line is the experimental Δ*H* while the red dotted lines indicate the 2 kcal/mol error limit. (b) Most populated conformations from each simulation for the 1DPU complex. The peptide is the helix at the top of the diagram. The blue–orange color scale represents the absolute difference between calculated and experimental Δ*H* in kcal/mol.

Although the initial results were quite poor in terms of correlation with the experiment, about half of the predictions were close to the experimental value, and this encouraged us to identify possible causes of error in the inaccurate results. First, 1DPU, where the receptor is the 32 kDa subunit of replication protein A (RPA32) gave an inaccurate Δ*H* with a difference of 7.12 kcal/mol between calculated and experimental values (Figure 3a, blue line). RPA32 interacts with DNA damage response proteins including SMARCAL1, Tipin, UNG2, and XPA.^32^ In the 1DPU structure, RPA32_(201–272)_ is in complex with a 73–88 residue long peptide from UNG2.

To identify potential problems with the Δ*H* calculation, we first performed clustering on the simulations of the complex using the single-linkage method with a 1 Å cutoff. We extracted the most populated conformations from each simulation. After superposition, the conformations were inspected and we observed highly flexible termini. Coloring conformations by the difference between calculated and experimental Δ*H* (Figure 3b), revealed two distinct conformations for the C-terminal, highly correlative with the experimental Δ*H* (Figure 3b). In one conformation (which we term **1DPU**_**tail2**_), good agreement is found with the experiment, and here, the terminal residue E270 interacts with K265 and K78 of UNG2. On the other hand, in the other conformation (**1DPU**_**tail1**_) (Figure S2a,b), E270 interacts with K217 and R88 of UNG2. This apparent correlation between conformations and binding enthalpies led us to perform additional simulations to increase the sampling and check the stability of the interaction between the terminal residue and K265. We then performed further simulations with 20 replicates using the **1DPU**_**tail2**_ conformation as the starting structure. We analyzed the distance between the C-terminal C atom and the side-chain N atom of residue K265 to check the stability of the interaction (Figure S2c). The second set of simulations revealed that the **1DPU**_**tail2**_ conformation is highly stable across 20 independent simulations. The most striking result to emerge from the binding enthalpy calculation is that the second set of simulations provided a noticeably more accurate Δ*H* (Figure 3a, orange line) than did the first simulation set. Thus, the C-terminal conformation of the receptor is not only flexible with two distinct conformations that interact with the peptide in distinct ways but also that one of these conformations is highly correlated with an accurate prediction of Δ*H*.

To further corroborate this finding, we considered a modified system, UNG2^RNK/AAA^, in which the peptide has three alanine mutations. ITC results have also been reported for this mutation.^32^ Importantly, this complex contains the K78A mutation, thus disrupting the interaction between E270 and K78 observed above. Nonetheless, the tail was quite stable in the **1DPU**_**tail2**_ conformation, as shown in Figure S2b despite the mutation (Figure S2c). To our surprise, the UNG2^RNK/AAA^ peptide also provided an accurate Δ*H* with the **1DPU**_**tail2**_ conformation (Figure S2d)—i.e., despite the removal of the key salt-bridge.

In all of these calculations so far, the apo-receptor simulations (see eq 1) were initiated from the **1DPU**_**tail1**_ conformation and as outlined above, good agreement with the experiment can be obtained when the complex simulations are initiated from the **1DPU**_**tail2**_ conformation (Figure S2). We explored this relationship further and performed additional simulations using only **1DPU**_**tail2**_ conformations for the apo-receptor component of the simulations to examine the effects on the Δ*H* prediction. The result was always worse when the receptor was initiated from the **1DPU**_**tail2**_ conformation in the apo-receptor simulations (Figure S2). Taken together, these findings suggest that the predominant conformational state of the apo-receptor differs from the preferred conformational state for the complex, at least in terms of the enthalpic component.

**Figure 4:**
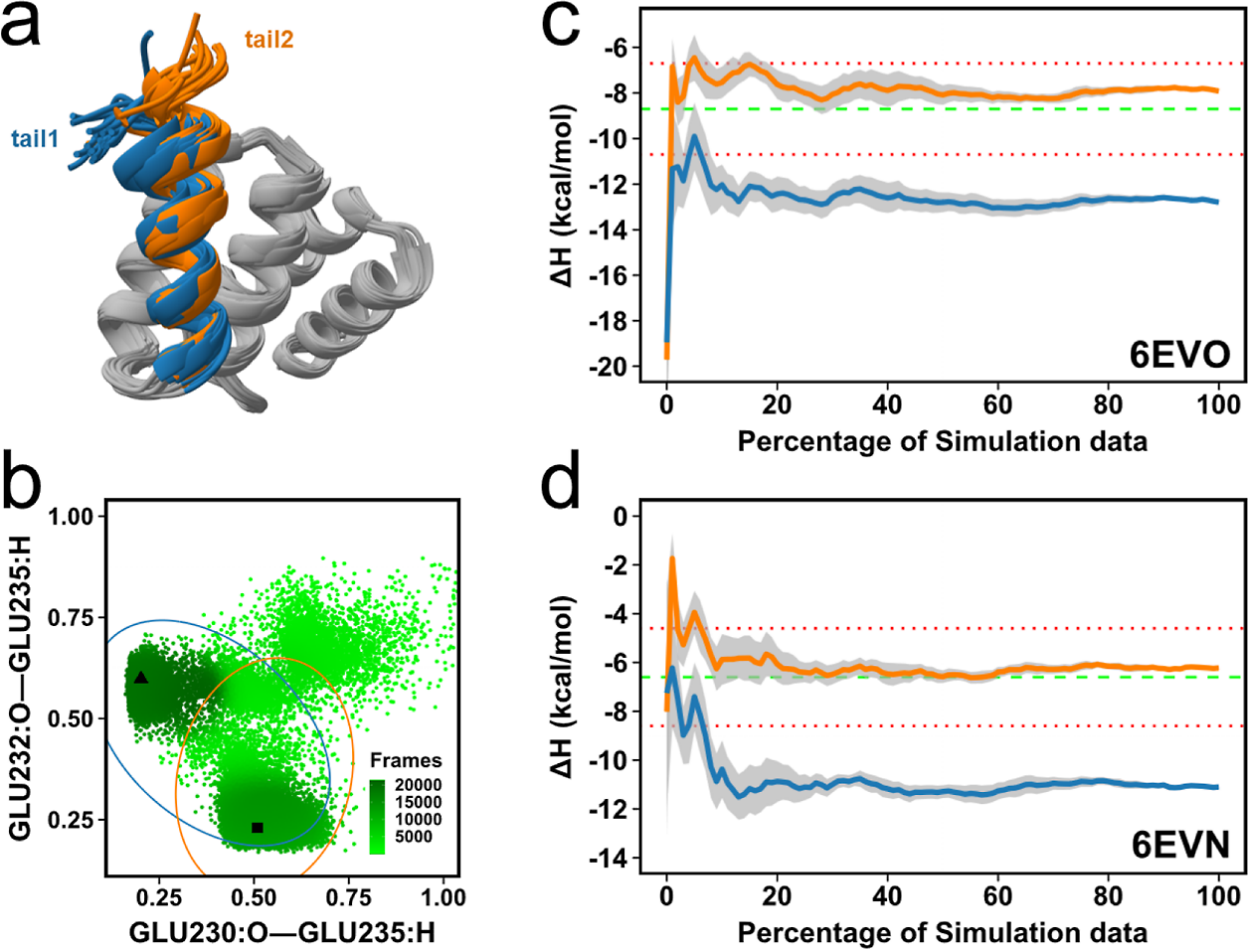
(a) Most populated conformations from each simulation for 40 apo-receptor simulations of two setups. (b) Convergence pattern of the calculated Δ*H* for 6EVO (c) distance (nm) distribution of two hydrogen bonds from apo-receptor simulations with two different tail setups. Elliptic circles show a 75% probability distribution for **tail1** (blue) and **tail2** (orange) simulations. The triangle shows hydrogen bond distances in the 6EVO crystal structure, while the square shows distances in 6EVM. (d) Convergence pattern of the calculated Δ*H* for 6EVN. For (b,d), the blue line shows calculated Δ*H* for the first simulation set (**tail1**), while the orange line shows for the second simulation set (**tail2**). The green dashed line is the experimental Δ*H*, while the red dotted lines indicate the 2 kcal/mol error limit.

The 6EVO complex also exists in two distinct tail conformations at the C-terminus. 6EVO contains the peptide-substrate-binding (PSB) domain of human type II collagen prolyl-4-hydroxylase (C-P4H-II) complexed with a proline-rich procollagen peptide (PPGPRGPPG).^41^ We found two different tail conformations at the C-terminal in both complex and apo-receptor simulations and also in two different crystal structures: 6EVO (**tail1**) and 6EVM (**tail2**) (Figures 4a and S3a). Like 1DPU (Figure S2), the tail contains negatively charged residues and these residues interact with positively charged residues around the tail (Figure S3b,c). In our initial calculations with 6EVO, the difference between the calculated and experimental enthalpy was 4.10 kcal/mol (Figure 4b, blue line). In the initial simulations, the tail mostly stayed in the **tail1** conformation. However, it was not clear how the **tail2** conformation would affect the enthalpy predictions.

Thus, we run an additional 20 independent simulations using the **tail2** conformation for both the complex and apo-receptor. Interestingly, the tail mostly remained stable with whatever conformation was used as the starting conformation (Figures 4c and S3f,g). We used two important backbone hydrogen bonds as a proxy for the tail’s conformational state. E235:H makes a hydrogen bond with E230:O in 6EVO, while it makes a different hydrogen bond with E232:O in 6EVM. This analysis revealed that the **tail1** conformation was more stable in the complex form than the apo-receptor (Figures 4b and S3h,i). We then checked the potential energies for each simulation setup. Complex simulations for protein–peptide did not give a significant difference with 1.41 kcal/mol for both simulation setups; on the other hand, apo-receptor simulations had very different potential energy profiles with 4.88 kcal/mol difference between **tail1** and **tail2** setups (Figure S3d,e). As a result, simulations of apo-receptor with **tail2** conformations improved the binding enthalpy prediction to a difference of 0.83 kcal/mol between calculation and experiment (Figure 4c, orange line).

To further confirm this finding, we performed an additional Δ*H* calculation for 6EVN having the same receptor complex with the PPGPAGPPG peptide. This peptide has an R5A mutation and −6.6 ± 0.1 kcal/mol binding enthalpy, as determined by ITC. Apo-receptor simulations with the **tail2** conformation provided more accurate Δ*H* than simulations starting from the **tail1** conformation (Figure 4d). Thus, overall, the results show that the receptor should have the **tail2** conformation in the apo state, but the **tail1** conformation in the bound state in order to obtain an accurate binding enthalpy calculation for the PSB domain of human C-P4H-II.

### Accurate Binding Enthalpy Hidden by Unknown Helix Formation

**Figure 5:**
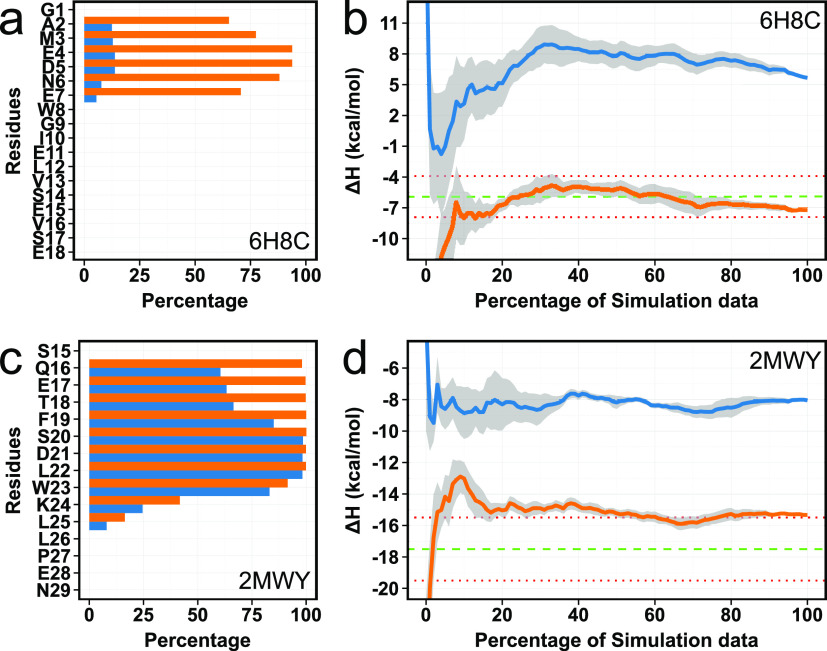
(a,c) α-Helix percentage per residue of the peptide. (b,d) Convergence pattern of the calculated Δ*H* (kcal/mol). The blue line shows calculated Δ*H* for the first simulation set, while the orange line shows for the second simulation set with helix formation. The green dashed line is the experimental Δ*H*, while the red dotted lines indicate the 2 kcal/mol error limit.

In two other outliers, we observed that a small α-helix had a tendency to form at the N-termini of the peptides (Figure 5). First, 6H8C is an NMR structure of the human GABARAPL2 protein in complex with the LIR motif that is found within UBA5 (ubiquitin-like modifier activating enzyme 5).^42^ Starting from the PDB coordinates of the complex gave us the worst initial Δ*H* prediction with the highest absolute difference of 11.78 kcal/mol between the calculated and experimental values (Table S1 and Figures 1b and 5b). However, only using trajectories where the helix was present in the complex resulted in a greatly improved enthalpy prediction (Figure S4a). This finding prompted us to conduct further simulations to increase the sampling of the helical conformation and test the stability of the helix. Thus, we ran additional 20 independent simulations using the complex with the helix present from the start. The secondary structure was computed for each simulation, and analysis revealed that the helix was relatively stable across almost all simulations (Figure 5a). Consequently, the second simulation set with the helix present dramatically improved the Δ*H* prediction (Figure 5b). However, we should be cautious in the interpretation here because the peptide used for the ITC measurements excluded the first 4 residues used in the NMR experiments (and also included 3 extra residues at the C-terminus)^42^ (Figure S4b). Thus, it is difficult to make a true direct comparison here.

The other complex where helix formation was observed was the MDMX-p53 complex from 2MWY. In this complex, the N-terminal domain of MDMX_(23–111)_ binds the first transactivation domain (TAD1) of p53_(15–29)_.^44^ When we looked at the experimental structures (Figure S5), the TAD1 peptide has a small helical structure, but an increase in the amount of helix of the TAD1 region was noticeable in our simulations. Interestingly, this same helix formation at the same region is also found in the AlphaFold model (https://alphafold.ebi.ac.uk/entry/P04637). This helix increase was clearly visible in >50% of the simulations (Figure 5c). We identified 10 simulations with the highest helix formation and examined their contribution to the binding enthalpy. While the simulations with the highest amount of helix improved the agreement with experimentally obtained enthalpy (−9.68 kcal/mol), the simulations with the least amount made the result even worse (−6.40 kcal/mol). Overall, the Δ*H* for 2MWY was estimated at −8.04 ± 0.08 kcal/mol for all simulations (Figures 2b and 5d). This suggested to us that once again, the presence of an additional helix in the peptide would improve the enthalpy prediction. Thus, we ran an additional 20 independent simulations for MDMX-p53 with the additional helical content. The secondary structure analysis revealed that the helix was highly stable across the simulations (Figure 5c), and the second simulation set with the helix formation significantly improved the Δ*H* prediction (Figure 5d).

### Metal Cation Parameters in Metalloproteins

In this data set, we had two metalloproteins; calmodulin having two Ca^2+^ ions (PDB: 2LQC) and the PHD zinc finger domain of BAZ2A that has two Zn^2+^ ions (PDB: 4Q6F) (Figure S6a). Calmodulin makes a complex with the “NSCaTE” peptide from the N-terminal cytoplasmic domain of the L-type voltage-gated calcium channel α1C subunit.^34^ This complex gave us an accurate Δ*H* with the absolute difference of 0.72 kcal/mol between calculated and experimental values (Figure S1 and Table S1) when we used the default parameters for Ca^2+^ within AMBER ff14SB. Given the known problems with calcium parametrization,^45^ this was a surprising result. On the other hand, the BAZ2A PHD zinc finger in complex with an unmodified H3K4 histone peptide in the 4Q6F structure provided an incorrect Δ*H* (Table S1 and Figure S6c) when using the default parameters for Zn^2+^ in AMBER ff14SB. Therefore, we investigated other parameters for Zn^2+^ and we ran an additional 40 simulations using the zinc AMBER force field (ZAFF)^46^ for the complex and apo-receptor simulations. The new setup improved the Δ*H* prediction with a 5.16 kcal/mol improvement for 4Q6F (Figure S6c) and placed just around the 2 kcal/mol error level.

### Considering Experimental Conditions

**Figure 6:**
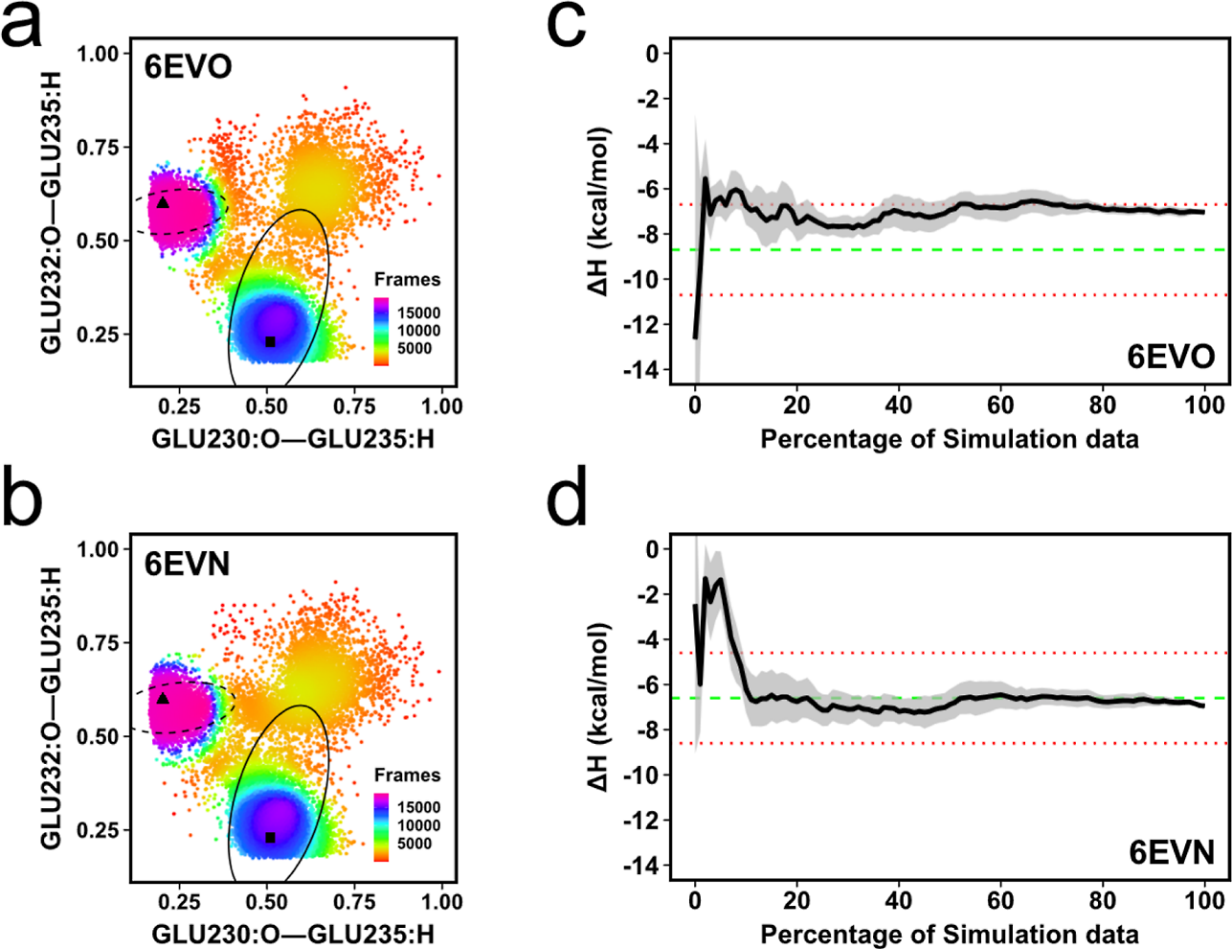
(a,b) Distance distribution of two hydrogen bonds from apo-receptor and complex simulations. Elliptic circles show a 75% probability distribution for complex (dashed) and apo-receptor simulations. The triangle shows hydrogen bond distances in the 6EVO crystal structure, while the square shows distances in 6EVM. (c,d) Convergence pattern of the calculated Δ*H* for 6EVO and 6EVN in experimental buffer conditions. The green dashed line is the experimental Δ*H*, while the red dotted lines indicate the 2 kcal/mol error limit.

We have performed all simulations with pure water to reduce complexity since we recently obtained good results for bromodomain–ligand complexes in pure water.^43^ The ITC experiments were mostly conducted under diverse buffer conditions (Table S2). Nevertheless, we wanted to see how accurate this approach is given an explicit buffer composition typical of that used in ITC experiments. We picked the PSB domain of human C-P4H-II (6EVN and 6EVO) since it required two different sampling approaches for complex and apo-receptor simulations. Thus, we set up additional simulations having 20 mM TRIS (tris(hydroxymethyl)aminomethane), 50 mM NaCl, and 50 mM glycine for the receptor–peptide complex and the apo-receptor. We used the **tail1** conformation in 6EVO for complex simulations as a starting conformation while the **tail2** conformation found in 6EVM was used for the apo-receptor simulations (Figure S3a) since this setup provided the most accurate results (Figure 4c,d). The same hydrogen bonds (Figure 4b) were used as a proxy to check the stability of the tail conformation (Figure 6a,b). The simulations show that an accurate Δ*H* prediction can be obtained whether simulating in pure water or a complete buffer condition for the PSB domain of human C-P4H-II (Figure 6c,d). Perhaps surprisingly, 6EVN gives a more accurate Δ*H* prediction in pure water than in buffer conditions (Figures 4c and 6d).

### Decompositions of Binding Enthalpies into Physical Components

Enthalpies obtained in this way from MD simulations can be further analyzed in terms of the energetic subcomponents.^15,47^ We investigated the determinants of the binding enthalpies from simulations, giving accurate results. Table 2 includes subcomponents of binding enthalpies for 11 protein–peptide systems into three physical determinants: changes in the van der Waals forces (**LJ**); changes in electrostatic interactions (**Coul**); changes in valence terms, which include bond-stretch, bond-angle, and dihedral terms (**Val**). The Lennard-Jones term was consistently negative for all complexes and makes a major contribution to the binding enthalpy. Unlike the Lennard-Jones contribution, the electrostatic contribution was mostly unfavorable, while valence terms varied in sign and magnitude. Interestingly, despite unfavorable contributions, there were moderate correlations between electrostatic contributions and experimental enthalpy values with 0.52 Pearson’s *r*. Hydrogen bonds (in the force field model employed here) are primarily electrostatic attractions between molecules. Thus, we checked for any correlations between enthalpy values and different aspects of the hydrogen bonds. We found modest correlations for the average total number of hydrogen bonds between the receptor and peptide with 0.48 Pearson’s *r* for experimental enthalpy values (Table S4). Perhaps surprisingly, there was no relationship between experimental enthalpy values and absolute hydrogen bond difference (i.e., all H-bonds in the system) between bound and unbound states (Δ*H* bond), but Δ*H* bond was highly correlated with **Val** (*r* = 0.73).

**Table 2:** Subcomponents of the Binding Enthalpies^a^

PDB	**Val**	**Coul**	**LJ**
1DPU	–1.80 ± 0.01	3.83 ± 0.14	–17.27 ± 0.09
1RST	0.01 ± 0.01	–1.45 ± 0.13	–8.62 ± 0.08
2LQC	2.92 ± 0.01	–0.66 ± 0.14	–9.89 ± 0.09
2MNU	–2.13 ± 0.01	7.33 ± 0.16	–9.00 ± 0.10
2MWY	3.95 ± 0.01	–2.39 ± 0.11	–16.89 ± 0.07
4F14	–0.49 ± 0.01	3.69 ± 0.14	–11.18 ± 0.09
4Q6F	–5.86 ± 0.01	12.24 ± 0.12	–14.05 ± 0.07
5E0M	–3.18 ± 0.01	10.75 ± 0.14	–14.20 ± 0.09
5OVC	–1.16 ± 0.01	8.90 ± 0.14	–16.46 ± 0.08
6EVO	2.34 ± 0.01	3.59 ± 0.12	–13.90 ± 0.07
6H8C	3.09 ± 0.01	8.83 ± 0.14	–19.15 ± 0.09

a **Coul**: Coulombic electrostatic contribution. **Val**: contribution from changes in bond-stretch, angle-bend, and dihedral terms. **LJ**: Lennard-Jones contribution. All values are in kcal/mol.

## Discussion

The combination of MD simulation, 3D structures, and experimental calorimetric data can provide a greater understanding of the driving forces for protein–protein binding.^7,48,49^ Ideally, one would like to be in a position to predict the absolute thermodynamic properties (Δ*G*, Δ*H*, and TΔ*S*) for any protein with its interaction, be that a small molecule or another protein, RNA, or DNA. Although much progress has been recently made on absolute Δ*G* predictions,^49^ it remains extremely challenging to do as well for Δ*H* calculations (though see ref (43) for some recent progress in this area) and progress has been modest. It is very challenging to do this in a prospective manner.

One possible reason for progress has been modest is the assumption that the amount of sampling required to ensure a converged result is simply too large and not currently obtainable. That is to some extent confirmed by this study whereby the initial results showed very poor agreement with the experimentally derived enthalpy values and indeed in some instances initiating calculations from one state does not move to a second conformational state, suggesting a significant energy barrier between the two states. One might well ask, what is the point of these calculations then?

There are several aspects to the answer. First, the lack of agreement for certain complexes suggests that their dynamical behavior is not correctly being captured or rather the simulation may be trapped in a conformational state that is not compatible with the enthalpy of that complex. Thus, if one is interested in the dynamics of a complex, the enthalpy prediction, if not in good agreement with the experiment, can be used as an indicator that there are likely to be other conformations that are not being sampled in the simulation. In this scenario, the use of any number of enhanced sampling techniques (such as metadynamics) could be usefully employed to improve the degree of conformational sampling. Thus, one can imagine a scenario where the ITC calculation is first performed, and if there is poor agreement then one would switch to metadynamics to improve the sampling and discover previously hidden but important states, but these are not necessarily a global panacea to the sampling problem. Many enhanced sampling methods require several order parameters, or collective variables (CVs),.^50^ However, if important CVs are missing, such methods may suffer from the “hidden energy barrier” problem and converge slowly.^51^ If no experimental or theoretical information is available, then one can use enhanced sampling methods without predefined reaction coordinates or CVs for binding enthalpy calculations. Such methods must accurately preserve both the thermodynamic and the kinetic properties of the system. However, their ability to reproduce accurate thermodynamic information has been questioned,^52^ and thus, more systematic investigation is still needed in this regard.

**Figure 7:**
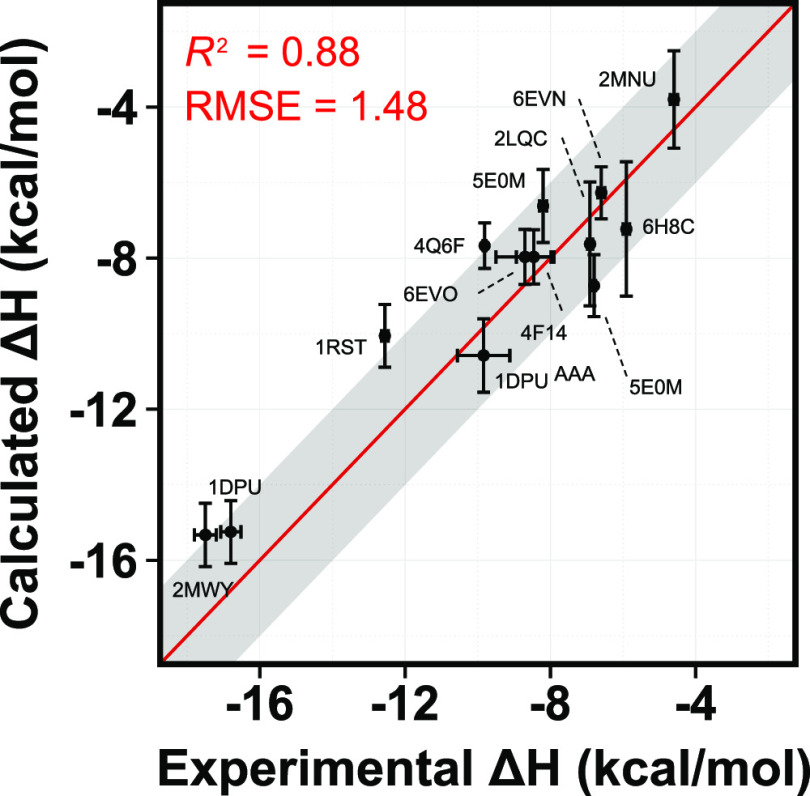
Comparison of calculated binding enthalpies from experimental values for 13 different protein–peptide systems (the 11 benchmarks plus 2 mutations). The line of equivalence is shown in red, and the gray shadow indicates the 2 kcal/mol error limit. Error bars were drawn by using the highest SEM values from the reblocking analysis.

Second, the work shows that if the correct conformations are being sampled well, then in fact the estimated enthalpies can be accurate. Although an artificial exercise, taking the best predictions from the simulations gives a very good agreement with the experiment (Figure 7). This shows that a commonly used force field (AMBER ff14SB) at least has the potential to predict enthalpies to a reasonable level. Of course, one should remember that ultimately the force field should predict Δ*G* and Δ*H* (and by inference TΔ*S*) correctly, and good performance in one aspect does not necessarily mean so for the other.

Accurate predictions (even posthoc corrected ones as summarized in Figure 7) may also be useful in terms of interpreting calorimetric calculations with atomistic details because calorimetric data often apparently contradict accepted scientific dogmas.^11,53,54^ For example, it was often thought that the enthalpic contribution of the binding is dominated by polar interactions in protein–ligand binding;^54,55^ however, the calculations here emphasize the importance of the van der Waals interactions for protein–protein systems (Table 2). Indeed the role of van der Waals interactions in stabilizing the protein–protein complexes has been the focus of recent studies.^56^ Moreover, a gain in the number of hydrogen bonds upon the binding was usually thought to be related to more favorable enthalpies, but we found no such correlation between overall change in Δ*H* bond and the binding enthalpy at least for these systems. These observations are intriguing and require further investigation, but a cursory inspection suggests that the peptide binding increases the number of vdW interactions.

Any calculations related to the free energy landscape of the binding between proteins will most likely be computationally very expensive since proteins can adopt many alternative conformations.^57^ Thus, insufficient conformational sampling is expected to hamper the accurate calculation of calorimetric components. Moreover, the free energy is a combination of many terms, which also increases the risks of errors and thus decreases precision and accuracy. Yet, a current meta-analysis of 853 studies from 34 different research groups showed that free energy methods were close to chemical accuracy (error = 1.58 kcal/mol).^58^ On the other hand, binding enthalpy–entropy calculations have generally not been given much consideration with MD simulations, and there are limited studies, especially for complex systems.^10,11,14,15,59^ It has been shown that an accurate binding enthalpy calculation is possible with a simplistic model, host–guest systems.^47,60,61^ Notable efforts have also been made for complex systems to obtain relative binding enthalpies.^11,14^ All of these studies have demonstrated that binding enthalpy calculations are sensitive to the choice of force field and sufficient sampling.

Previously, we reported a comprehensive assessment of force fields and water models using a set of 25 cucurbit[7]uril–guest (CB7) pairs and showed that the TIP3P water model combination with general AMBER force field provided good agreement with the experimental enthalpy values.^61^ From the sampling perspective, it is unclear how much simulation data is needed to obtain accurate binding enthalpies of complex systems. Roy et al. ran 40 independent 10 ns simulations to get sufficient sampling with 1 ps writing frequency for energy data.^14^ Furthermore, Li and Gilson reached over 250 μs of simulation time by seeding every 200 ns and recording energies every 2 ps.^11^ So far, these were the only examples using the direct method for complex systems from the literature. Here, we performed 20 completely independent repeats of 100 ns simulations and recorded energy data every 100 fs for each system, and as discussed above, inaccurate enthalpies came mostly from incorrect global sampling exemplified by conformational states not seen experimentally.

It is well-known that receptors can adopt alternative conformations upon peptide binding,^62^ as reported here. Here, we observed the C-terminal loop region having two major conformations in RPA32 (Figure 3b) and the PSB domain of human C-P4H-II proteins (Figure 4a). In the NMR structure (1DPU), RPA32 exhibits conformational heterogeneity in the C-terminal, but in the simulations, two distinct conformations were populated (Figure S2c), and RPA32 provided accurate enthalpies for UNG2^WT^ and UNG2^RNK/AAA^ peptides only when it had two different conformations in bound and unbound states. Likewise, the PSB domain of human C-P4H-II also provided accurate enthalpies for two different peptides (6EVO and 6EVN) in pure water and experimental buffer conditions (20 mM Tris, 50 mM NaCl, and 50 mM glycine) when having two different conformations in bound and unbound states. Conversely, the PSB domain has these conformations in X-ray crystal structures (Figure S3a): **tail1** conformation in 6EVN, 6EVO, and 6EVP, **tail2** conformation in 6EVL (apo) and 6EVM, which contains a weak binder (P)_9_ peptide. These suggest that the C-terminal of the PSB domain tends to have the **tail1** conformation with good binders but the **tail2** conformation with weak binders or when in the apo form. This observation of conformational differences is mirrored in our simulations and enthalpy calculations. Interestingly, there are charged residues around the C-terminal in both cases of the RPA32 (Figure S2a,b) and PSB domains of human C-P4H-II (Figure S3b,c), and it is possible that they drive the conformational change via electrostatic interactions. We also note that, paradoxically, the choice of small systems to aid the sampling problem could also have meant that termini near the interface could be more prone to setup dependencies.

In contrast to large folded domains, peptides are usually in the form of unstructured/disordered molecules in the unbound state.^63^ They sometimes undergo folding upon binding, which results in better structural adaptation with a much lower level of conformational freedom. For example, the Bak peptide gets an α-helix when complexed to Bcl-x_L_, although it is an unstructured random coil in solution.^64^ Importantly, free p53 TAD, which we used in our benchmark as a peptide (2MWY), is intrinsically disordered. Furthermore, NMR studies showed that the p53 TAD did not tend to create a helical structure in the free form.^65^ However, the p53 TAD gets more helical upon binding to MDM2, MDMX,^66,67^ and the nuclear receptor coactivator binding domain of CREB binding protein.^68^ These structural stabilizations are concomitant with a large enthalpic gain.^69^ In the same way, we observed large enthalpic gains in two different protein–peptide systems upon helix formation in MDM2-p53 (2MWY) and GABARAPL2-UBA5 (6H8C; Figure 5). The p53-MDM2/MDMX complexes have been well-characterized as a target for cancer therapy.^44,67,70^ The UBA5 peptide (^337^EDNEWGIELVSEVSE^351^) is an unstructured region in crystal structures and the AlphaFold model,^71^ but it formed a small helix at the N-terminal in our simulations. Consequently, complex simulations having more helix formation in the peptides provided more accurate results for the binding enthalpies of the MDM2-p53 and GABARAPL2-UBA5 complexes. Interestingly, there is an artificial cloning artifact (GAM) in the ITC construct of the UBA5 peptide (Figure S4b). Thus, this artificial extension in the N-terminal possibly drives helix formation in the UBA5 peptide upon binding. On the other hand, the p53 TAD was already a helix in the PDB structure, but it was shorter than we observed in the simulations and the AlphaFold model (Figure S5). Similarly, more helix formation ensured more accurate binding enthalpy for the MDM2-p53 complex as well. At this point, it is also worth remembering that the underlying tendency of the force field to adopt helical conformations is an aspect that has received attention in recent years^72^ and that force field development is an ongoing exercise.^73^

Considering complete experimental conditions in simulations might increase the complications during setup and computational time. The choice of the buffer can affect the thermodynamic signatures if there are proton movements between solute and buffer during binding.^74^ Moreover, it has been suggested that buffer salts might increase the hydration of the ligands on the binding of agonists and antagonists at the histamine H3 receptor.^75^ Nonetheless, the buffer conditions did not appear to dramatically affect the enthalpy calculations in these calculations since we got highly accurate calculations with simulations in pure water (Figure 7). In addition, we reported accurate binding enthalpies for bromodomain–ligand complexes in pure water after considering ZA-loop dynamics.^43^ On the contrary, Gao et al. reported the strong sensitivity of the computed binding enthalpies to the concentration of NaCl (0–500 mM) for the CB7-B2 system.^47^ As was discussed,^74^ this may be attributed to cation binding at the portals CB7 that are displaced upon binding of the guest. Although strong sensitivities to salt concentrations were observed in calculations with different water models, there was no significant effect of the experimental buffer condition according to our observations. Here, we reported similar results for binding enthalpies of the PSB domain of human C-P4H-II with two different peptides (6EVO and 6EVN) in pure water and also experimental buffer conditions (20 mM TRIS, 50 mM NaCl, and 50 mM glycine). Although we observe no real effect of buffer in this study, this is an area that requires a more systematic and complete approach that is beyond the scope of this paper. It should not be forgotten that differences between the simulations and ITC conditions can also contribute significantly to the overall error.

More than 50% of proteins include metal ions, making them prevalent in biological systems.^76^ Metalloproteins can perform vital tasks like scavenging free radicals and catalyzing respiration reactions in the cell.^77^ Consequently, two metalloproteins were included in our benchmark; calmodulin having two Ca^2+^ (PDB: 2LQC) and the PHD zinc finger domain of BAZ2A having two Zn^2+^ (PDB: 4Q6F). 2LQC gave us an accurate Δ*H*, while 4Q6F provided an incorrect Δ*H* when we used the default parameters in AMBER ff14SB. Here, the nonbonded model, the simplest model among nonpolarizable models, was used for metals.^78^ It consists only of the electrostatic and van der Waals terms. However, it was not reproducible for the hydration-free energy and ion–oxygen distance of the first solvation shell for the zinc ion when using the nonbonded model.^22,79^ Later, Li and Merz proposed bonded models for different zinc-coordinated systems (ZAFF), which reproduced the experimental hydration-free energy, coordination number, and ion–oxygen distance simultaneously.^22,23,80^ Obtaining better results after using ZAFF pointed out that further improvement in force field accuracy for Zn^2+^ was needed, as might be expected.

## Conclusions

In conclusion, we have shown that the predicted enthalpy is a straightforward way to assess whether a protein–peptide system is likely to sample the correct ensemble of states. Furthermore, posthoc explicit consideration of this in further simulations suggests that at least the AMBER force field used here is capable of producing enthalpy values in agreement with the experiment. However, we are still some way off from being able to perform these calculations prospectively, and to that end our assembled data set of diverse protein–peptide complexes complete with binding enthalpy–entropy data could serve as a valuable benchmark for the community. Such benchmarks are extremely useful for drug discovery studies by providing opportunities for comparing algorithms.^16,81^ The data set is available at doi: http://10.0.20.161/zenodo.7635945, including the PDB IDs and ITC details. It should be an invaluable resource for computational structural biologists trying to predict thermodynamic components from the structure and to test new methods for protein–protein interactions.

## Data Availability

Complete input and parameter files, along with ITC summary details are available at doi: http://10.5281/zenodo.7635945.

## Supporting Information Available

Breakdown of potential energies, experimental buffer conditions for each system, enthalpies for additional calculations and detailed hydrogen bond analysis; individual convergence patterns, details of specific tail interactions, detailed conformational analysis of specific systems, and pictorial representations of hydrogen-bond analysis (PDF)
