# Supplementary material for: Computed Protein–Protein Enthalpy Signatures as a Tool for Identifying Conformation Sampling Problems
Source: J Chem Inf Model. 2023 Sep 28;63(19):6095–108. doi: 10.1021/acs.jcim.3c01041 (PMC10565830; doi:10.1021/acs.jcim.3c01041)
Supplement: Supplementary file 1 — ci3c01041_si_001.pdf [file ci3c01041_si_001.pdf]

SUPPORTING INFORMATION

Computed protein-protein enthalpy signatures as a tool for identifying conformation sampling problems.

Süleyman Selim Çınaroğlu and Philip C. Biggin

Department of Biochemistry, University of Oxford, South Parks Road, Oxford, OX1 3QU, UK

*To whom correspondence should be addressed.
philip.biggin@bioch.ox.ac.uk

ORCID 0000-0001-5100-8836

Keywords: protein structure, enthalpy, thermodynamics, protein-protein interactions

TABLES

Table S1: *Initial binding enthalpy values for each PDB, and their potential energies for all simulation setups.*

PDB	ΔH_{EXP}	ΔH_{CAL}	COMPLEX	RECEPTOR	PEPTIDE	WATER
1DPU	−16.80 ± 0.28	-9.75 ± 0.97	-109091.26 ± 0.67	-107733.26 ± 0.42	-97202.80 ± 0.51	-95854.55 ± 0.22
1RST	−12.56 ± 0.09	-10.06 ± 0.83	-155956.57 ± 0.63	-155575.17 ± 0.43	-48298.92 ± 0.27	-47927.58 ± 0.21
2LQC	−6.91 ± 0.07	-7.63 ± 1.64	-122705.54 ± 1.17	-122100.59 ± 1.04	-96451.86 ± 0.43	-95854.55 ± 0.22
2MNU	−4.60 ± 0.10	-3.80 ± 1.29	-194074.04 ± 0.80	-193324.03 ± 0.73	-96600.76 ± 0.66	-95854.55 ± 0.22
2MWY	−17.50 ± 0.30	-8.04 ± 1.14	-107800.93 ± 0.92	-107200.34 ± 0.33	-48520.13 ± 0.55	-47927.58 ± 0.21
4F14	–8.46 ± 0.48	-7.97 ± 0.72	-100078.30 ± 0.33	-100023.01 ± 0.56	-95901.86 ± 0.22	-95854.55 ± 0.22
4Q6F	−9.81 ± 0.04	-2.51 ± 1.37	-123636.04 ± 0.90	-123001.41 ± 0.99	-48559.70 ± 0.22	-47927.58 ± 0.21
5E0M	–8.20 ± 0.10	-6.62 ± 0.97	-102729.31 ± 0.57	-102119.96 ± 0.50	-96457.28 ± 0.55	-95854.55 ± 0.22
5OVC	−6.80 ± 0.04	-8.73 ± 0.82	-128689.74 ± 0.68	-128196.94 ± 0.34	-48411.65 ± 0.21	-47927.58 ± 0.21
6EVO	−8.70 ± 0.80	-12.80 ± 0.83	-110970.19 ± 0.53	-110750.40 ± 0.58	-48134.57 ± 0.18	-47927.58 ± 0.21
6H8C	−5.91± 0.09	5.66 ± 2.41	-117771.75 ± 1.77	-117017.16 ± 1.44	-96614.80 ± 0.75	-95854.55 ± 0.22

All values are in kcal/mol. The uncertainties were calculated using blocking analysis while potential energy values using ensemble averaging.

Table S2: *Experimental buffer conditions of ITC experiment for each PDB entries*

PDB	ITC Condition	Ref.
1DPU		Xie et al.[1]
1RST	50 mM Potassium Phosphate, pH 7.6	Schmidt et al.[2]
2LQC	20 mM HEPES, 100 mM KCl, and 5 mM CaCl2, pH 7.0	Liu et al.[3]
2MNU	20 mM Sodium Phosphate, pH 6.0	Yu et al.[4]
2MWY	10 mM Sodium Phosphate, 200 mM NaCl and 1 mM TCEP, pH 6.5	Grace et al.[5]
4F14	Tris 12.5 mM, NaCl 150 mM, pH 8.0	Eulitz et al.[6]
4Q6F	20 mM HEPES, 150 mM NaCl, 0.5 mM TCEP, pH 8.0	Tallant et al.[7]
5E0M	50 mM Sodium Phosphate, 50 mM NaCl, 0.5 mM NaN_3, pH 7.5	Clark et al.[8]
5OVC	50 mM HEPES, 150 mM NaCl, pH 7.0	Ponna et al.[9]
6EVO	20 mM TRIS, 50 mM NaCl, and 50 mM Glycine, pH 8.0	Murthy et al.[10]
6H8C	50 mM TRIS, 100 mM NaCl, pH 7.5	Huber et al.[11]

Table S3: *Binding enthalpy values for additional calculations, and their potential energies for all simulation setups.*

PDB	ΔH_{EXP}	ΔH_{CAL}	COMPLEX	RECEPTOR	PEPTIDE	WATER
1DPU_tail	−16.80 ± 0.28	-15.25 ± 0.83	-109096.76 ± 0.44	-107733.26 ± 0.42	-97202.80 ± 0.51	-95854.55 ± 0.22
1DPU_AAA	-9.84 ± 0.28	-10.58 ± 0.97	-108821.20 ± 0.48	-107733.26 ± 0.42	-96931.90 ± 0.70	-95854.55 ± 0.22
2MWY_helix	−17.50 ± 0.30	-15.33 ± 0.84	-107808.23 ± 0.51	-107200.34 ± 0.33	-48520.13 ± 0.55	-47927.58 ± 0.21
4Q6F_zaff	−9.81 ± 0.04	-7.67 ± 0.60	-122372.79 ± 0.30	-121733.00 ± 0.43	-48559.70 ± 0.22	-47927.58 ± 0.21
6EVO_tail	−8.70 ± 0.80	-7.97 ± 0.73	-110970.19 ± 0.53	-110755.24 ± 0.42	-48134.57 ± 0.18	-47927.58 ± 0.21
6H8C_helix	−5.91± 0.09	-7.23 ± 1.78	-117784.64 ± 0.72	-117017.16 ± 1.44	-96614.80 ± 0.75	-95854.55 ± 0.22
6EVN	−6.60 ± 0.80	-11.10 ± 0.80	-110791.47 ± 0.46	-110750.40 ± 0.58	-47957.55 ± 0.21	-47927.58 ± 0.21
6EVN_tail	−6.60 ± 0.80	-6.27 ± 0.69	-110791.47 ± 0.46	-110755.24 ± 0.42	-47957.55 ± 0.21	-47927.58 ± 0.21
6EVO_ion	−8.70 ± 0.80	-7.04 ± 1.26	-112488.62 ± 1.09	-112274.59 ± 0.57	-48134.57 ± 0.18	-47927.58 ± 0.21
6EVN_ion	−6.60 ± 0.80	-6.95 ± 0.93	-112311.51 ± 0.67	-112274.59 ± 0.57	-47957.55 ± 0.21	-47927.58 ± 0.21

All values are in kcal/mol.
The uncertainties were calculated using blocking analysis while potential energy values using ensemble averaging.
_ion: 20 mM TRIS (tris(hydroxymethyl)aminomethane), 50 mM NaCl, and 50 mM glycine in the solution
_zaff: Zinc AMBER Force Field (ZAFF) for Zn^{2+}
_tail: considering alternative tail conformations
_helix: considering helix formation in the peptide
_AAA: the peptide has RNK/AAA mutation.

Table S4: *The solvent-accessible surface area (SASA) for bound and unbound states. Hydrophobic portions of SASA for bound (HB) and unbound (HU) states. Average number of hydrogen bonds between receptor and peptide (HBonds). HBA: average number of hydrogen bonds per SASA change between bound (HB) and unbound (HU) states. ∆HBond; absolute hydrogen bond difference between bound and unbound states.*

PDB ID	Bound (nm^2)	Unbound (nm^2)	HB (%)	HU (%)	HBonds	HBA	∆HBond
1DPU	61.70	73.83	31.32	37.54	6.87	0.57	-0.65
1RST	81.35	90.75	44.47	49.58	5.24	0.56	0.40
2LQC	64.27	77.44	32.92	41.42	4.83	0.37	1.22
2MNU	69.79	85.39	36.94	46.70	8.04	0.52	1.51
2MWY	64.91	80.04	32.52	41.41	4.14	0.27	2.82
4F14	52.00	62.81	24.93	31.66	4.67	0.43	0.66
4Q6F	52.52	60.26	24.25	31.31	8.12	1.05	-2.57
5E0M	63.35	73.51	28.90	33.92	14.60	1.44	1.23
5OVC	61.11	71.63	32.09	37.71	13.37	1.27	0.66
6EVO	62.68	73.19	30.27	35.80	8.50	0.81	0.70
6H8C	80.41	99.25	38.67	50.34	11.89	0.63	1.59

All calculated by using simulation sets giving good agreement with experimental for the binding enthalpies.

FIGURES

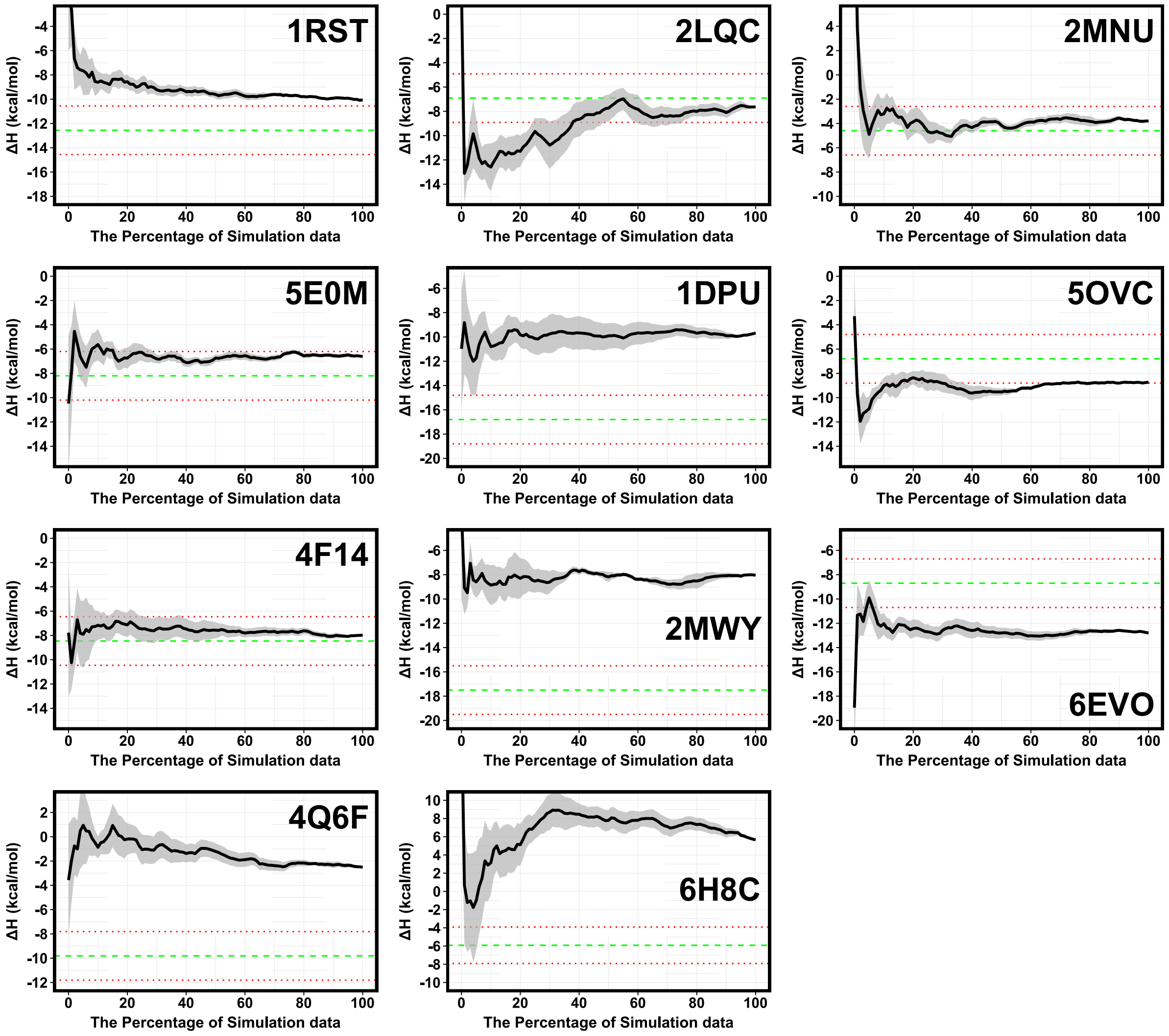

Figure S1: *Convergence pattern of the calculated ΔH by using 60 trajectories from the initial simulations of each PDB. Green dashed-line is the experimental ΔH while red dotted-lines indicate the 2 kcal/mol error limit.*

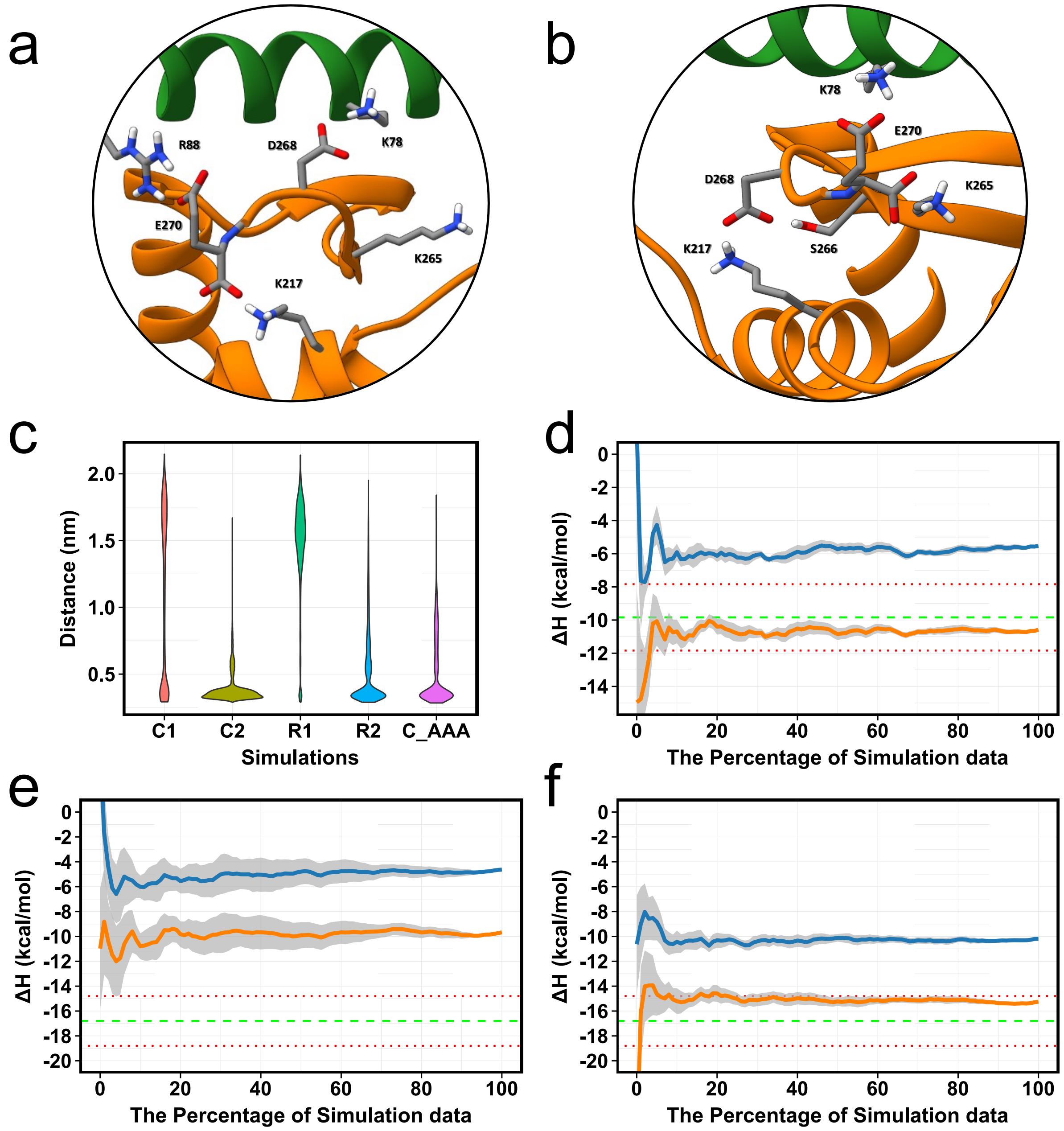

Figure S2: ***a-b)*** *Residues around the tail and their interactions in two conformations extracted from simulations; 1DPU$_{tail1}$ in* ***a*** *and 1DPU$_{tail2}$ in* ***b****.* ***c)*** *The violin plots for the distance between C-terminal C atom and side-chain N atom of the residue K265. C1 and R1 are initial simulations for complex and apo-receptor, respectively. C2 and R2 are additional simulations having more sampling of the second tail conformation in b.* ***d)*** *Convergence pattern of the calculated ΔH by using C_AAA with R1 (orange) and R2 (blue) simulations. Peptide has a mutation (RNK/AAA) in C_AAA simulations.* ***e)*** *Convergence pattern of the calculated ΔH by using C1 with R1 (orange) and R2 (blue) simulations.* ***f)*** *Convergence pattern of the calculated ΔH by using C2 with R1 (orange) and R2 (blue) simulations.*

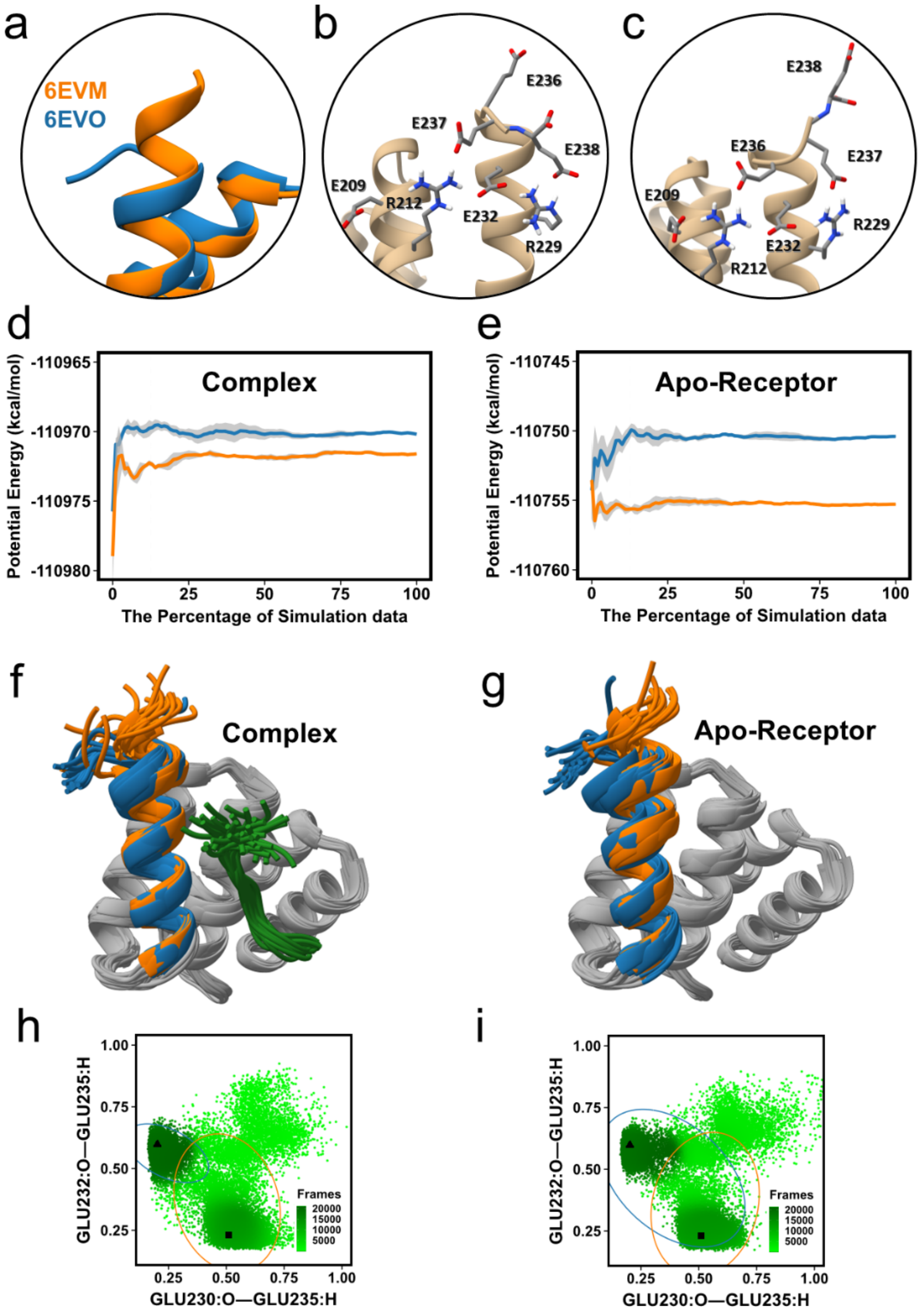

Figure S3: ***a)*** *The tail conformations in crystal structures.* ***b-c)*** *Residues around the tail and their interactions in two conformations extracted from simulations.* ***d-e)*** *Convergence plots of the potential energy for complex and apo-receptor simulations having two different tail conformations.* ***f-g)*** *The most populated conformations from each simulation set.* ***h-i)*** *Distance (nm) distribution of two hydrogen bonds from apo-receptor and complex simulations with two different tail setups. Elliptic circles show 75% probability distribution for tail1 (blue) and tail2 (orange) simulations. Triangle shows hydrogen bond distances in 6EVO crystal structure while square shows distances in 6EVM.*

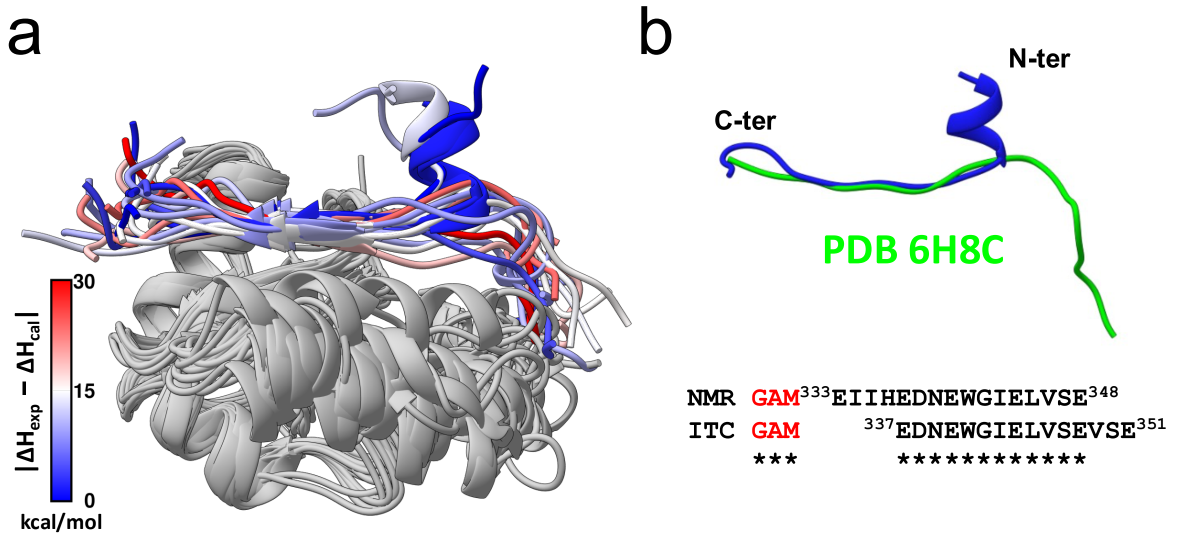

Figure S4: ***a)*** *The most populated conformations from each simulation for complex simulations. Blue-Red color scale represents the absolute difference between calculated and experimental ΔH.* ***b)*** *Superimposition of the peptide from the NMR structure and the simulation. Sequence alignment of the peptide used in NMR and ITC experiment. The first three residues (GAM) are due to a cloning artefact*[11]*.*

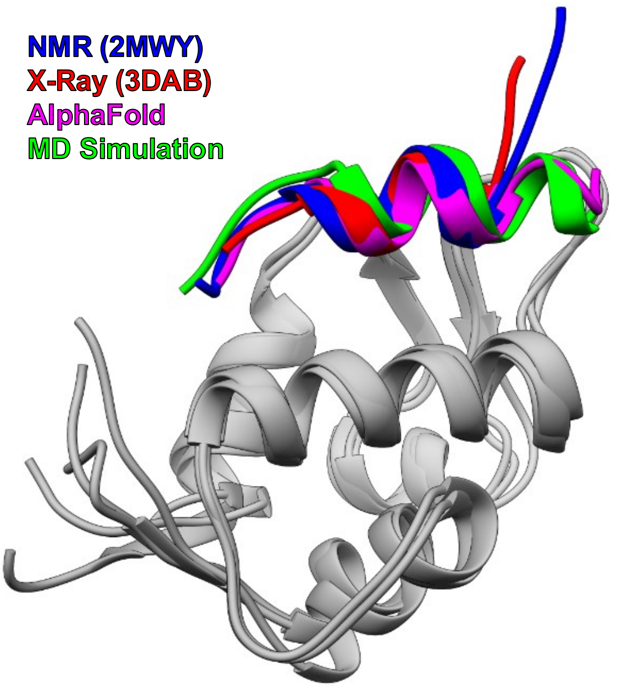

Figure S5: *Superimposition of three different MDMX-P53 complexes. Blue is NMR structure (PDB: 2MWY) which we used in the study. Red is X-Ray structure (PDB: 3DAB). Green was observed in the simulations. Magenta: Alphafold model obtained from https://alphafold.ebi.ac.uk/entry/P04637*

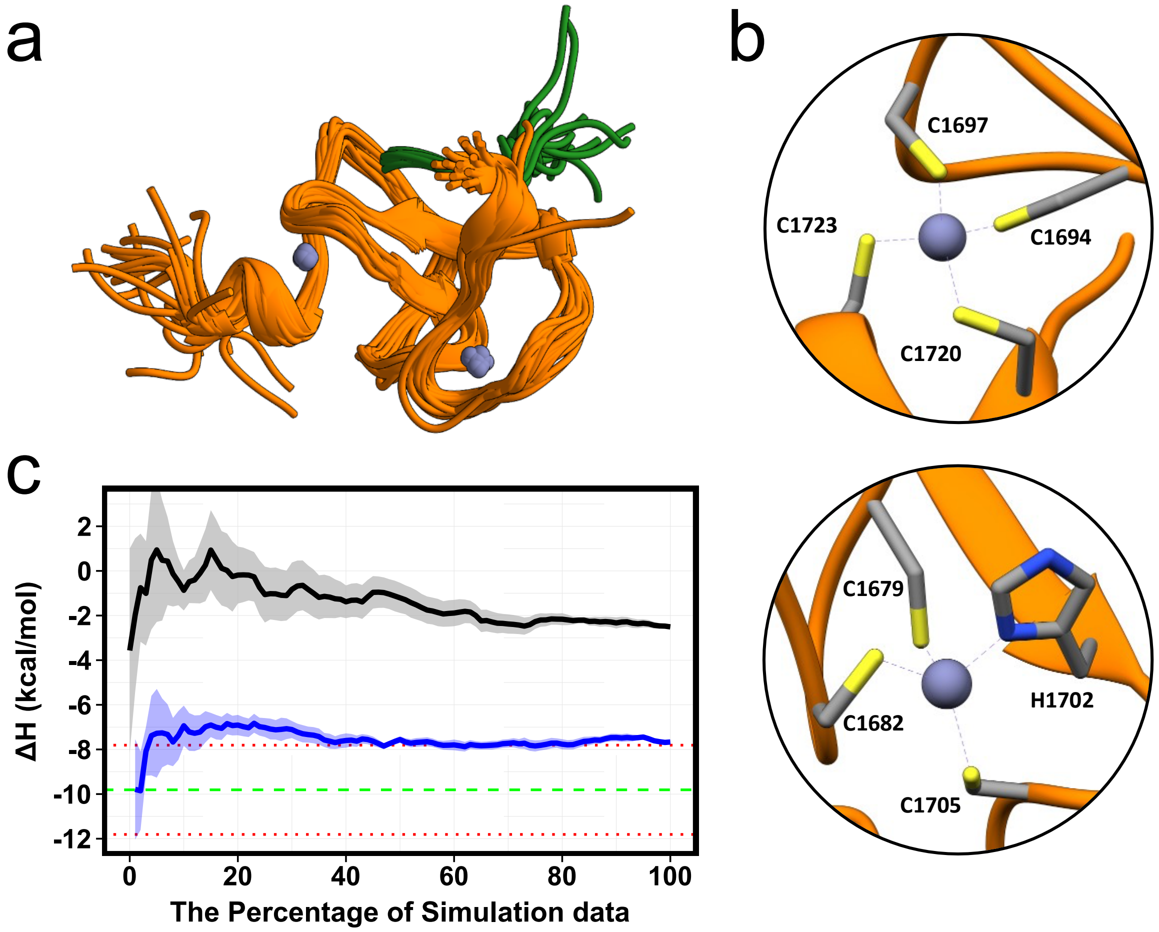

Figure S6: ***a)*** *The most populated conformations from each simulation for complex simulations.* ***b)*** *Zn metal centers and surrounding residues in the receptor. different MDMX-P53 complexes.* ***c)*** *Convergence pattern of the calculated ΔH by using default* Zn^{2+} *parameters in Amber FF14SB (black) and Zinc AMBER force field (ZAFF) (blue)*

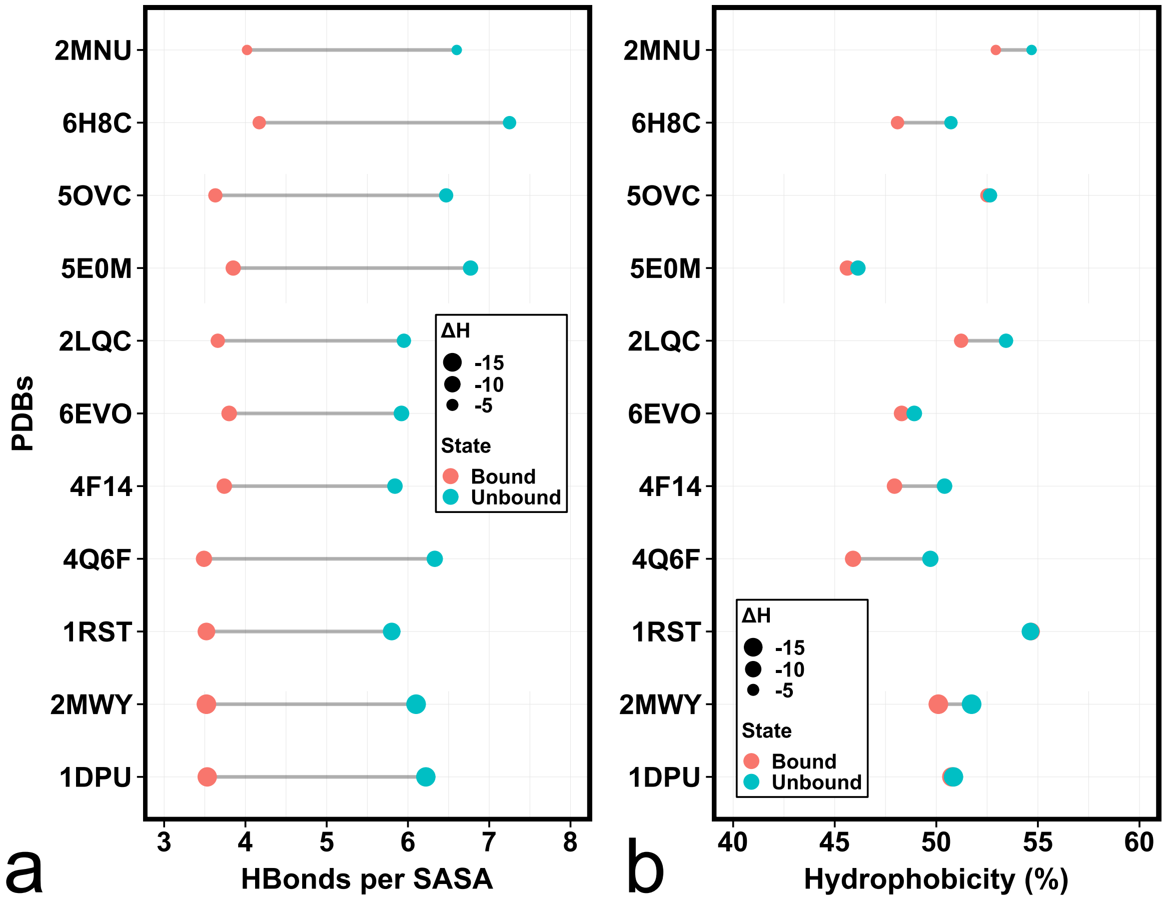

Figure S7: ***a)*** *The number hydrogen bonds per SASA between solute and solvent.* ***b)*** *Overall change in hydrophobic area between unbound and bound states. PDB IDs were sorted based on experimental ΔH values.*

REFERENCES

(1) Xie, S.; Lu, Y.; Jakoncic, J.; Sun, H.; Xia, J.; Qian, C. Structure of RPA 32 bound to the N‐terminus of SMARCAL 1 redefines the binding interface between RPA 32 and its interacting proteins. *FEBS J.* **2014**, *281* (15), 3382-3396.

(2) Schmidt, T. G.; Koepke, J.; Frank, R.; Skerra, A. Molecular interaction between the Strep-tag affinity peptide and its cognate target, streptavidin. *J.Mol. Biol.* **1996**, *255* (5), 753-766.

(3) Liu, Z.; Vogel, H. J. Structural basis for the regulation of L-type voltage-gated calcium channels: interactions between the N-terminal cytoplasmic domain and Ca2+-calmodulin. *Front. Mol. neurosci.* **2012**, *5*, 38.

(4) Yu, T. K.; Shin, S. A.; Kim, E. H.; Kim, S.; Ryu, K. S.; Cheong, H.; Ahn, H. C.; Jon, S.; Suh, J. Y. An unusual protein–protein interaction through coupled unfolding and binding. *Angew. Chemie., Int. Ed.* **2014**, *126* (37), 9942-9945.

(5) Grace, C. R.; Ban, D.; Min, J.; Mayasundari, A.; Min, L.; Finch, K. E.; Griffiths, L.; Bharatham, N.; Bashford, D.; Guy, R. K. Monitoring ligand-induced protein ordering in drug discovery. *J. Mol. Biol.* **2016**, *428* (6), 1290-1303.

(6) Eulitz, S.; Sauer, F.; Pelissier, M.-C.; Boisguerin, P.; Molt, S.; Schuld, J.; Orfanos, Z.; Kley, R. A.; Volkmer, R.; Wilmanns, M. Identification of Xin-repeat proteins as novel ligands of the SH3 domains of nebulin and nebulette and analysis of their interaction during myofibril formation and remodeling. *Mol. Biol. Cell.* **2013**, *24* (20), 3215-3226.

(7) Tallant, C.; Valentini, E.; Fedorov, O.; Overvoorde, L.; Ferguson, F. M.; Filippakopoulos, P.; Svergun, D. I.; Knapp, S.; Ciulli, A. Molecular basis of histone tail recognition by human TIP5 PHD finger and bromodomain of the chromatin remodeling complex NoRC. *Structure* **2015**, *23* (1), 80-92.

(8) Clark, S.; Nyarko, A.; Löhr, F.; Karplus, P. A.; Barbar, E. The anchored flexibility model in LC8 motif recognition: insights from the chica complex. *Biochemistry* **2016**, *55* (1), 199-209.

(9) Ponna, S. K.; Ruskamo, S.; Myllykoski, M.; Keller, C.; Boeckers, T. M.; Kursula, P. Structural basis for PDZ domain interactions in the post‐synaptic density scaffolding protein Shank3. *J. Neurochem.* **2018**, *145* (6), 449-463.

(10) Murthy, A. V.; Sulu, R.; Koski, M. K.; Tu, H.; Anantharajan, J.; Sah‐Teli, S. K.; Myllyharju, J.; Wierenga, R. K. Structural enzymology binding studies of the peptide‐substrate‐binding domain of human collagen prolyl 4‐hydroxylase (type‐II): High affinity peptides have a PxGP sequence motif. *Prot. Sci.* **2018**, *27* (9), 1692-1703.

(11) Huber, J.; Obata, M.; Gruber, J.; Akutsu, M.; Löhr, F.; Rogova, N.; Güntert, P.; Dikic, I.; Kirkin, V.; Komatsu, M. An atypical LIR motif within UBA5 (ubiquitin like modifier activating enzyme 5) interacts with GABARAP proteins and mediates membrane localization of UBA5. *Autophagy* **2020**, *16* (2), 256-270.
